# Impact of Micro and Nanoplastics on Reproductive Cancer and the Potential Anticancer Benefits of Prolonged Ginger, Garlic, and Turmeric Consumption: A Narrative Review

**DOI:** 10.3390/ijerph23040471

**Published:** 2026-04-07

**Authors:** Babatunde Adebola Alabi, Onyemaechi Okpara Azu, Zodwa Dlamini, Richard Khanyile, Rahaba Marima

**Affiliations:** 1 Pan African Cancer Research Institute (PACRI), SAMRC Precision Oncology Research Unit (PORU), Oncology and Cancer Prevention, University of Pretoria, Prinshof, Pretoria 0028, South Africa; zodwa.dlamini@up.ac.za; 2 Department of Medical Oncology, Faculty of Health Sciences, Steve Biko Academic Hospital, University of Pretoria, Prinshof, Pretoria 0002, South Africa; richard.khanyile@up.ac.za; 3 Department of Medical Biosciences, University of the Western Cape, Bellville 7535, South Africa; oazu@uwc.ac.za

**Keywords:** environmental carcinogenesis, garlic, ginger, microplastics, nanoplastics, reproductive cancer, turmeric

## Abstract

**Highlights:**

**Public health relevance—How does this work relate to a public health issue?**

- Human exposure to micro- and nanoplastics (MP/NPs) is extremely widespread, with evidence of accumulation in biological systems, raising concerns about their role in key biological processes like oxidative stress, inflammation, endocrine disruption, and genotoxicity, which are central to reproductive health disorders and cancer development.
- This review connects these reproductive toxicological mechanisms with the potential modulatory effects of widely consumed dietary phytochemicals from ginger, garlic, and turmeric, which are known to influence similar molecular pathways.

**Public health significance—Why is this work of significance to public health?**

- The study highlights a critical gap between rising MP/NP exposure and the limited understanding of its long-term reproductive and carcinogenic risks in humans, while proposing biologically plausible mitigation strategies.
- It integrates emerging evidence suggesting that bioactive compounds in ginger, garlic, and turmeric may counteract oxidative stress, inflammation, and genotoxicity, key processes implicated in MP/NP-related toxicity.

**Public health implications—What are the key implications or messages for practitioners, policy makers and/or researchers in public health?**

- While reducing environmental exposure remains essential, promoting diets rich in antioxidant and anti-inflammatory phytochemicals (e.g., ginger, garlic, and turmeric) may offer a complementary, low-risk strategy to mitigate potential health effects.
- Further research is needed to validate these protective effects in the context of MP/NP exposure, particularly through well-designed epidemiological and clinical studies assessing long-term dietary intake and health outcomes.

**Abstract:**

Human exposure to micro- and nanoplastics (MP/NPs) is increasingly recognized as a potential environmental health concern, although their role in reproductive carcinogenesis remains unclear. This narrative review aims to evaluate current evidence linking MP/NP exposure to reproductive cancers and to explore the potential chemoprotective effects of bioactive compounds derived from ginger, garlic, and turmeric. A structured literature search was conducted using PubMed, Scopus, Web of Science, and Google Scholar for studies published between 2008 and 2026. Relevant in vitro, in vivo, and human biomonitoring studies were included to assess mechanisms of toxicity, while preclinical and clinical studies were reviewed to examine the anticancer properties of selected dietary phytochemicals. Available evidence suggests that MP/NPs can accumulate in human biological systems, including reproductive tissues, where they induce oxidative stress, chronic inflammation, endocrine disruption, and DNA damage, processes closely associated with carcinogenesis. Although epidemiological data remain limited and do not establish cancer, emerging biomonitoring and experimental findings support a biologically plausible link between MP/NP exposure and hormone-related cancers. Concurrently, bioactive compounds such as curcuminoids, gingerols, and organosulfur compounds demonstrate the ability to modulate key molecular pathways involved in oxidative stress, inflammation, and cell proliferation. Preclinical studies consistently report anticancer effects, while early clinical evidence suggests improvements in oxidative and inflammatory biomarkers, though definitive therapeutic benefits remain uncertain. Overall, this review highlights important mechanistic links and identifies dietary phytochemicals as potential modulators of MP/NP-induced carcinogenic pathways. However, further well-designed epidemiological and clinical studies are needed to clarify causal relationships and validate their protective role.

## 1. Introduction

Plastic, valued for its affordability, versatility, and chemical stability, has seen rapid global expansion, with annual production increasing. In 2020 alone, global plastic production was estimated at 367 million metric tons, of which only 9% was recycled, and projections indicate it may reach 1.2 billion tons by 2060 [1,2]. Human exposure to plastics occurs primarily through oral ingestion, inhalation, and dermal contact, with ingestion recognized as the predominant route [3,4]. Plastics accumulate in ecosystems due to their persistence and limited recyclability, posing significant environmental and health hazards [5]. During use and disposal, plastics undergo fragmentation and degradation via physical, chemical, and biological processes, including mechanical abrasion, photodegradation, thermal oxidation, and biodegradation, forming smaller plastic fragments [6].

Plastics that degrade into particles measuring 0.1–5000 μm are termed microplastics, whereas those measuring 0.001–0.1 μm are referred to as nanoplastics [7,8,9]. Major commercial plastics include polypropylene (PP), polyethylene (PE), polyethylene terephthalate (PET), polystyrene (PS), and polyvinyl chloride (PVC). Based on origin, microplastics are categorized as either primary (industrial particles or microbeads produced for specific applications) or secondary, which arise from the environmental degradation of larger plastic debris through photodegradation, abrasion, or water erosion [10,11]. These materials appear as fibers, granules, fragments, or films and are further classified by chemical composition into PET, high-density polyethylene (HDPE), low-density polyethylene (LDPE), PVC, PP, and PS.

Plasticizers, commonly used as additives, have been identified as endocrine-disrupting chemicals (EDCs) [12,13]. MP/NPs and their associated plasticizers can interfere with key signaling pathways of endocrine glands, including the pituitary, thyroid, adrenal, and gonads, thereby disrupting hormone regulation and essential metabolic functions related to homeostasis, fertility, neural development, and fetal growth [14]. Due to their nanoscale dimensions, micro- and nanoplastics can penetrate reproductive cells, tissues, and organs, disturbing the morphology and physiology of both male and female systems [15,16]. Their interaction with sex hormone receptors further raises concerns regarding their potential role in the onset and progression of hormone-responsive malignancies such as cervical, breast, ovarian, endometrial, testicular, and prostate cancers [17,18]. Although current clinical and preclinical studies remain limited, emerging evidence supports these concerns. Microplastics have been detected in ovarian follicular fluid from women undergoing assisted reproduction, highlighting potential reproductive exposure pathways [19]. Similarly, increased microplastic exposure levels have been reported in patients with progressive cervical cancer, providing direct evidence of a possible association between plastic exposure and carcinogenic risk [20]. While these studies cannot establish causality, the detection of microplastics in human reproductive tissues underscores the need for systematic evaluation of MP/NPs as potentially modifiable environmental risk factors for reproductive cancers.

MP/NPs can infiltrate tissues and interact with steroid receptors, including estrogen, androgen, thyroid hormone, progesterone, and peroxisome proliferator-activated receptors, leading to hormonal dysregulation, oxidative stress, chronic inflammation, and DNA damage, all of which are plausible mechanisms of tumorigenesis in hormone-sensitive organs [21]. MP/NP exposure may disrupt major oncogenic and tumor-suppressive pathways (WNT/β-catenin, PI3K/Akt/mTOR, MAPK/ERK, NF-κB, and p53), promoting proliferation, inflammatory signaling, impaired apoptosis, and genomic instability [22,23]. Considering these potential pro-carcinogenic mechanisms, ROS generation, immune modulation, and co-transport of carcinogens, together with the ubiquity of plastic exposure, a systematic evaluation of epidemiological and experimental evidence linking MP/NPs to reproductive cancer risk is urgently needed.

Similarly, dietary bioactive agents with recognized anticancer properties warrant exploration as potential chemopreventive interventions. Ginger, garlic, and turmeric, widely consumed worldwide, contain bioactive compounds such as gingerols/shogaols, allicin, and curcumin, which exhibit anti-inflammatory, antioxidant, and antiproliferative effects in preclinical studies [24]. These natural agents may modulate molecular pathways implicated in MP/NP-driven carcinogenesis and therefore represent promising candidates for mitigating related health risks.

Therefore, the objective of this narrative review is to critically evaluate current epidemiological and experimental evidence on the potential role of MP/NPs in the development and progression of reproductive cancers. In addition, this review aims to examine the mechanistic pathways through which these particles may promote carcinogenesis, including endocrine disruption, oxidative stress, inflammatory signaling, and key oncogenic pathway dysregulation. Furthermore, the review explores the potential chemopreventive and anticancer properties of prolonged dietary consumption of ginger, garlic, and turmeric, with particular emphasis on their bioactive phytochemicals and their capacity to modulate molecular pathways implicated in plastic-associated carcinogenic processes.

The increasing global burden of plastic pollution has raised concerns about chronic human exposure to MP/NPs and their potential health implications. Emerging evidence suggests that these particles can accumulate in biological tissues, disrupt endocrine signaling, and induce oxidative stress and inflammatory pathways that may contribute to carcinogenesis in hormone-sensitive organs [25]. However, current knowledge remains fragmented, with limited integrative analyses examining the potential link between MP/NP exposure and reproductive cancers. Moreover, there is growing interest in identifying dietary phytochemicals capable of mitigating environmentally induced carcinogenic processes. Therefore, a comprehensive evaluation of available evidence is necessary to clarify the potential role of MP/NPs in reproductive cancer risk and to explore the possible chemopreventive benefits of bioactive compounds derived from ginger, garlic, and turmeric.

### 1.1. Literature Search Strategy

A comprehensive literature search was conducted to identify relevant studies examining the biological and toxicological effects of micro- and nanoplastics on reproductive systems and cancer-related mechanisms. Scientific databases, including PubMed, Google Scholar, Scopus, and Web of Science, were searched for peer-reviewed articles published primarily between 2008 and 2026. Search terms included combinations of keywords such as “microplastics,” “nanoplastics,” “reproductive toxicity,” “oxidative stress,” “inflammation,” “genotoxicity,” “reproductive cancer,” “endocrine disruption,” and “phytochemicals.” Additional relevant studies were identified through manual screening of the reference lists of selected articles. Priority was given to recent studies, mechanistic investigations, and experimentally validated findings, including in vitro studies, animal models, and available human biomonitoring studies. Because this article is a narrative review, the objective was to evaluate and critically discuss emerging evidence rather than to conduct a formal systematic review or meta-analysis.

### 1.2. Inclusion and Exclusion Criteria

Inclusion criteria: peer-reviewed articles, studies investigating MP/NP exposure or plastic-associated chemicals (e.g., BPA, phthalates), and outcomes related to oxidative stress, endocrine disruption, genotoxicity, or cancer (in vitro, in vivo, and clinical studies). Exclusion criteria: non-English publications, conference abstracts without full data, studies lacking mechanistic or health-related endpoints, and studies focusing solely on environmental distribution without biological relevance.

## 2. Toxicological Mechanisms of Micro and Nanoplastics (MP/NPs) in Reproductive Tissues (Ovarian, Cervical, Prostate, Testicular)

Current understanding of *MP/NP* biological effects on reproductive tissues is derived from a combination of mechanistic evidence from experimental animal models, in vitro, and human biomonitoring studies. While the detection of microplastics in human biological samples has revealed widespread environmental exposure, much of the mechanistic evidence for their potential toxicity and carcinogenicity was obtained from cell line and animal studies. These experimental models provide important insights into the molecular and cellular pathways through which MP/NPs may affect reproductive health, including oxidative stress, chronic inflammation, endocrine disruption, and genotoxic damage.

### 2.1. Mechanistic Evidence from Preclinical Studies

#### 2.1.1. Induction of Oxidative Stress

MP/NPs can infiltrate reproductive organs, disrupting cellular activities and exerting toxic effects on reproductive tissues primarily through the induction of oxidative stress [26]. MP/NPs bind to intracellular organelles, particularly mitochondria, triggering excessive production of reactive oxygen species (ROS) [26,27]. Elevated ROS levels induced by MP/NP exposure promote lipid peroxidation and increase intracellular peroxynitrite formation, thereby exacerbating oxidative damage to lipids, proteins, and nucleic acids. Although such oxidative stress responses have been reported across various cell types exposed to MP/NP*s*, reproductive tissues may be particularly susceptible due to their high metabolic activity, endocrine sensitivity, and the vulnerability of germ cells to oxidative damage. Consequently, these processes can impair cellular homeostasis in the gonads and accessory reproductive organs.

**Figure 1:**
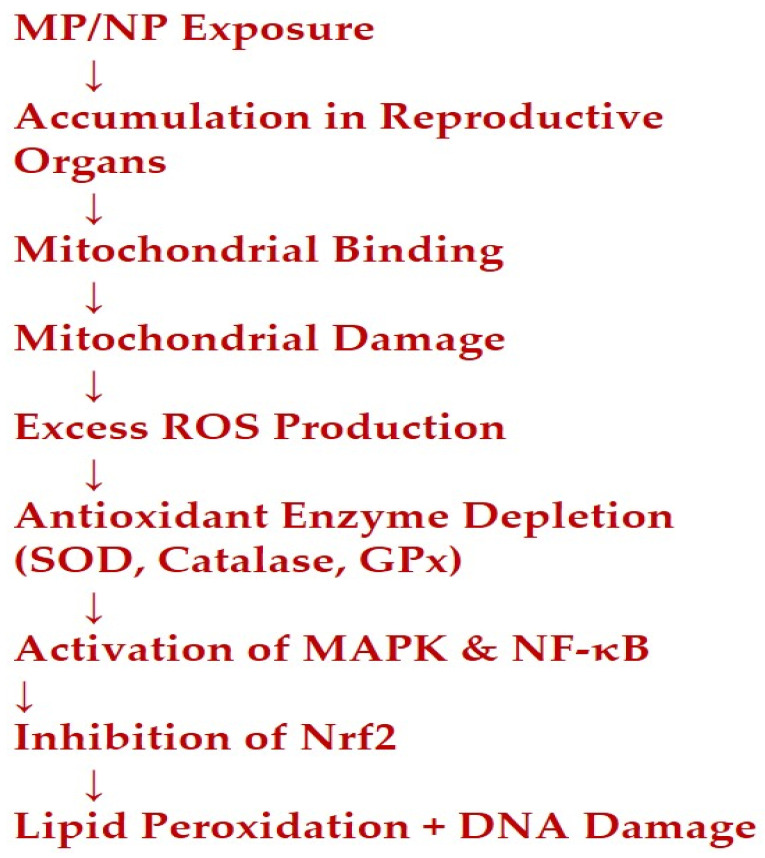
Oxidative-stress-mediated mechanisms of MP/NP-induced reproductive toxicity. MP/NPs accumulate in reproductive tissues and interact with intracellular organelles, particularly mitochondria. This interaction disrupts mitochondrial structure and membrane potential, leading to excessive production of reactive oxygen species. Increased oxidative stress results in lipid peroxidation and accumulation of peroxynitrite and malondialdehyde while depleting antioxidant enzymes such as superoxide dismutase, catalase, and glutathione peroxidase. These oxidative changes activate MAPK and NF-κB signaling pathways while suppressing Nrf2-mediated antioxidant responses, culminating in DNA damage and cellular dysfunction.

Several studies in rat and aquatic models have demonstrated that MP/NPs deplete key antioxidant enzymes, including superoxide dismutase, catalase, and glutathione peroxidase, leading to elevated accumulation of peroxynitrite and malondialdehyde [28,29]. Mitochondrial swelling, damaged cristae, and loss of membrane potential in germ cells and follicular cells often accompany these biochemical disruptions (Figure 1). In female rat models, MP/NP exposure increases tissue levels of free radicals while attenuating cytoplasmic antioxidant capacity, leading to impaired oocyte maturation [26,30,31]. Similar effects occur in testicular tissues, where spermatogenic cells exhibit vacuolation, chromatin condensation, and reduced sperm motility [26,32].

Oxidative stress induced by MP/NPs also activates the MAPK signaling pathway and the NF-κB inflammatory transcription factor, while inactivating the antioxidant transcription factor Nrf2, cellular responses aimed at counteracting the oxidative insult [23]. Chronic MP/NP exposure and persistent oxidative stress will eventually overwhelm the antioxidant defense mechanisms, leading to extensive DNA damage [27]. Evidence across species indicates that oxidative stress acts as a key initiator of MP/NP-induced reproductive toxicity, frequently interacting with inflammatory, endocrine, and genotoxic pathways [26].

#### 2.1.2. Activation of Chronic Inflammation and Immune Modulation

**Figure 2:**
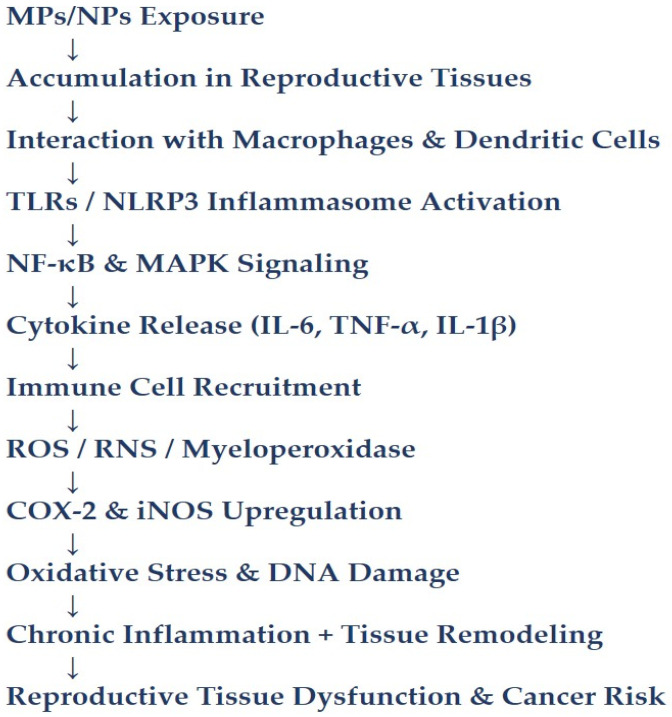
Mechanistic pathways of MP/NP-induced inflammation and oxidative stress in reproductive tissues. MP/NPs accumulate in reproductive tissues where they interact with macrophages and dendritic cells, activating toll-like receptors and the NLRP3 inflammasome. This stimulates NF-κB and MAPK signaling pathways, resulting in the release of pro-inflammatory cytokines such as IL-6, TNF-α, and IL-1β. Cytokine signaling promotes immune cell recruitment and amplifies inflammation, leading to increased production of reactive oxygen and nitrogen species, upregulation of COX-2 and iNOS, oxidative damage, tissue remodeling, and disruption of reproductive homeostasis.

Exposure to MP/NPs has been shown to induce inflammatory responses through oxidative-stress-mediated mechanisms and activation of pro-inflammatory signaling pathways. Although acute inflammatory responses may vary depending on exposure conditions, accumulating evidence suggests that MP/NP exposure can promote persistent low-grade inflammation and immune dysregulation, thereby disrupting immune homeostasis in reproductive tissues [33,34]. MP/NPs interact with macrophages and dendritic cells in the gonadal microenvironment, stimulating the release of pro-inflammatory cytokines such as interleukin-6, tumor necrosis factor-alpha, and interleukin-1β [35]. These cytokines act as chemotactic agents, recruiting additional immune cells and exacerbating local inflammation and tissue remodeling. Further immune cell infiltration enhances the release of myeloperoxidase and reactive oxygen species (ROS), amplifying oxidative damage (Figure 2).

In the testes, chronic inflammation compromises the integrity of the blood–testis barrier, leading to degeneration of seminiferous tubules, infiltration of macrophages and lymphocytes into interstitial spaces [36]. Comparable immune activation patterns occur in the ovary, mammary gland, and uterus, marked by upregulated expression of cyclooxygenase-2 and inducible nitric oxide synthase, which further promote the production of ROS and reactive nitrogen species (RNS) [37,38,39,40]. The persistent presence of MP/NPs in reproductive tissues can also trigger immune recognition receptors, including toll-like receptors (TLRs) and the NLRP3 inflammasome complex, thereby amplifying inflammatory signaling pathways [23,41].

MP/NP-induced chronic inflammation does not occur in isolation; it is closely intertwined with endocrine and metabolic disturbances. Inflammatory microenvironments foster fibrotic changes and impair tissue regeneration, particularly in ovarian stroma and testicular interstitium. Continuous immune activation in these tissues can increase the risk of DNA damage.

#### 2.1.3. Hormonal Disruption Through Endocrine Interference

Prolonged exposure to MP/NPs in reproductive tissues sustains oxidative stress and chronic inflammation, ultimately leading to endocrine interference and hormonal disruption [42]. This can be referred to as an indirect endocrine-disrupting effect of plasticizers. MP/NPs can also directly mimic natural hormones by binding to estrogen or androgen receptors, resulting in an eccentric activation or antagonism [43,44,45]. These interactions alter receptor-mediated gene transcription and promote abnormal cellular proliferation. Endocrine effects are not confined to gonadal tissues but also involve hypothalamic and pituitary dysfunction, reflecting broader systemic disruption of hormonal regulation. MP/NPs disrupt the hypothalamic–pituitary–gonadal (HPG) axis, impairing key reproductive hormone synthesis, secretion, and receptor-mediated actions [46,47,48,49].

Preclinical studies have shown that exposure to MP/NPs reduces serum concentrations of gonadotropins and sex steroids, including the luteinizing hormone, follicle-stimulating hormone, testosterone, and estradiol [50]. In male rodent models, low levels of testosterone in serum correlate with suppressed expression of steroidogenic enzymes such as StAR, CYP11A1, and 17β-HSD. In female rats, impaired estrogen synthesis is associated with disrupted aromatase activity and downregulation of follicular FSH receptor expression [21,51,52]. These hormonal imbalances ultimately compromise gametogenesis, ovulation, and implantation.

#### 2.1.4. DNA Damage and Genotoxic Effects Leading to Carcinogenesis

Prolonged exposure to MP/NPs induces excessive production of reactive species and chronic inflammation, both of which are key pathways contributing to DNA strand breaks, base-pair mismatches, and chromosomal aberrations [53,54,55]. In addition, nanoplastics can directly interact with chromatin granules and other intranuclear genetic materials, thereby disrupting DNA replication and transcription. Collectively, these mechanisms highlight the genotoxic potential of MP/NPs and their capacity to cause DNA damage in reproductive cells, forming the basis of their potential carcinogenicity.

**Figure 3:**
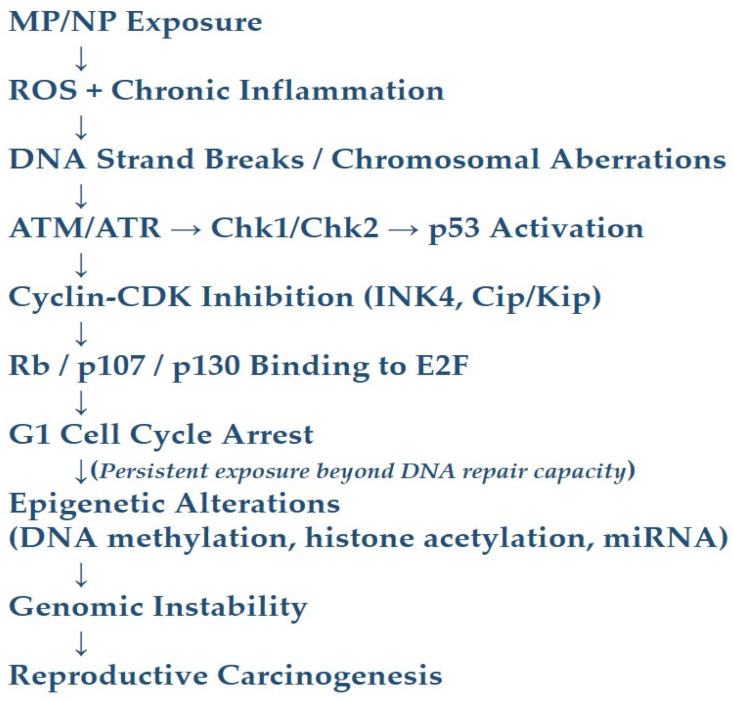
Genotoxic and cell-cycle regulatory mechanisms induced by MP/NP exposure in reproductive cells. Prolonged exposure to MP/NPs promotes excessive generation of reactive oxygen and nitrogen species, and chronic inflammatory responses, leading to DNA strand breaks, base-pair mismatches, and chromosomal aberrations. Nanoplastics may also directly interact with chromatin structures, disrupting DNA replication and transcription. DNA damage activates cellular checkpoint pathways involving ATM/ATR, Chk1/Chk2, and p53 signaling, which inhibit cyclin-dependent kinase activity through INK4 and Cip/Kip proteins and promote binding of Rb family proteins to E2F transcription factors, resulting in cell-cycle arrest. Persistent exposure may overwhelm DNA repair mechanisms and induce epigenetic alterations like DNA methylation changes, histone acetylation imbalance, and microRNA dysregulation, ultimately contributing to genomic instability and reproductive carcinogenesis.

In vitro studies on ovarian and testicular cell lines reveal increased comet tail length, micronucleus formation, and disrupted cell cycle progression after MP/NP exposure [56,57]. Activation of cyclin-dependent kinase inhibitors gene and enhanced expression of INK4 proteins can lead to G1 arrest by promoting redistribution of Cip/Kip proteins and blocking cyclin E-CDK2 activity. Retinoblastoma (Rb), p107, and p130, which are pocket proteins, are upregulated and bind to the E2F transcription factors to prevent progression past the G1 checkpoint. DNA damage triggers activation of Chk2 or Chk1, p53, and the ATM/ATR pathway to arrest the cell cycle and repair damaged genes at G1 and G2 phases. However, persistent exposure to nanoplastics can overwhelm the capacity of DNA repair mechanisms (Figure 3), leading to mutation and genomic instability [58]. Epigenetic alterations represent another means by which MP/NPs induce genotoxicity [59,60]. Exposure has been linked to alterations in DNA methylation and histone acetylation, as well as the dysregulation of microRNAs that control genes involved in cell proliferation, apoptosis, and differentiation.

The convergence of oxidative, inflammatory, and genotoxic pathways provides a mechanistic link between MP/NP exposure and reproductive carcinogenesis (Figure 3). The preclinical models show that long-term exposure to MP/NPs can promote cellular proliferation and inhibit apoptosis, hallmarks of tumor initiation. In addition, the endocrine-disrupting properties of MP/NPs and their additives can stimulate hormone-responsive signaling pathways, such as estrogen receptor activation, which are relevant in the etiology of ovarian, endometrial, prostate, and testicular cancer.

### 2.2. Evidence from Human Studies

Although much of the mechanistic understanding of MP/NP toxicity has been derived from experimental and in vitro studies, emerging human biomonitoring investigations provide important evidence that exposure to these particles occurs in real-world populations (Table 1). Recent studies have detected microplastics in several human biological matrices, including; blood, placental tissue, lung samples, and reproductive fluids, suggesting that systemic distribution and tissue accumulation may occur following chronic environmental exposure [61,62].

The presence of microplastics in human blood indicates that these particles can circulate within the body and potentially reach distant organs through the vascular system [3]. Similarly, the detection of microplastics in placental tissues highlights the possibility of maternal–fetal transfer and raises concerns regarding potential reproductive and developmental effects [63]. Evidence of microplastic contamination in lung tissues further supports the notion that inhalation represents an important route of human exposure [64]. Notably, recent reports describing the presence of microplastics in reproductive-associated biological fluids, such as ovarian follicular fluid, suggest that these particles may directly interact with reproductive cells during critical stages of gamete development [19].

Despite these findings, it is important to emphasize that current human studies largely document exposure and tissue presence rather than direct causal relationships with disease outcomes. Consequently, while biomonitoring evidence supports the biological plausibility of MP/NP accumulation in human tissues. Definitive causal links between MP/NP exposure and reproductive carcinogenesis in humans are yet to be established. Future research involving large-scale epidemiological studies, longitudinal cohort analysis, and improved exposure assessment methods will be essential to clarify the long-term health implications of chronic MP/NP exposure in human populations.

#### Human Epidemiological Data Linking Environmental MP/NP Exposure to Reproductive Cancers

There is a paucity of human epidemiological data on exposure to MP/NPs and reproductive cancer risk, but studies are gradually emerging [66]. A study conducted by Xu et al. [20] found that the level of exposure to MP/NPs also increases during the development of cervical cancer. Recent findings suggest that low serum levels of BPA may be associated with cancer development and may influence responses to cytotoxic therapy [67,68,69]. There are inconsistencies in evidence from epidemiological studies on the effect of phthalates on breast cancer incidence. A study examining the implications of urinary concentrations of nine phthalate metabolites found that exposure to diethyl phthalate, the parent compound of monoethyl phthalate (MEP), may be associated with increased risk of breast cancer [70]. In contrast, evidence from this study also revealed an inverse association between exposure to the parent compounds of monobenzyl phthalate (MBzP) and mono(3-carboxypropyl) phthalate (MCPP) and breast cancer risk, suggesting that higher exposure was linked to a decreased likelihood of developing breast cancer [71].

These discrepancies may partly reflect variations in study populations, exposure assessment methods, and sample sizes, as well as differences in the extent to which potential confounders such as age, lifestyle factors, endocrine disorders, and co-exposure to other environmental toxicants were controlled. Importantly, the detection of MP/NPs in human biological samples, including blood, placenta, semen, and ovarian tissue, provides direct evidence of internal exposure. While these findings do not establish causality, they demonstrate that MP/NPs are bioavailable and can reach critical reproductive sites. However, the external validity of these findings remains uncertain because many biomonitoring studies are conducted in geographically restricted populations [72], limiting the generalizability of results across different environmental and socio-economic contexts.

Preliminary reports suggest a correlation between environmental plastic exposures and altered reproductive functions. Reduced sperm quality [73], menstrual irregularities [74], and hormonal imbalances [75] were shown to be associated with consistent and long-term exposure to plasticizers. Nevertheless, many of these studies rely on observational or cross-sectional designs that may be vulnerable to selection bias, recall bias, and residual confounding from lifestyle and occupational exposures. Although only a limited number of studies have directly investigated cancer outcomes, similarities with known endocrine-disrupting chemicals provide biological plausibility for a potential link between MP/NP exposure and reproductive malignancies.

Some studies reported a high burden of microplastics in tumor tissues compared to adjacent non-tumor tissues, suggesting potential involvement in tumor initiation or progression [76,77]. Another study analyzing paired prostate tumor and paratumor tissues identified and quantified microplastics in both sample types [78]. The analysis demonstrated that microplastics were present in tumor tissues and differed in abundance and characteristics compared with nearby non-tumor tissue, suggesting potential interactions between microplastics and tumor microenvironments. A study analyzing 61 tumor samples across different cancer types confirmed the presence of microplastics within tumor tissues and proposed that these particles may influence the tumor immune microenvironment and treatment response [79]. Also, it has been reported that colorectal cancer tissues contained a greater diversity and distribution of microplastics compared with adjacent non-tumor tissues, indicating possible preferential accumulation within malignant tissues [80].

Epidemiological investigations in this field, therefore, face substantial methodological challenges, including exposure misclassification due to evolving analytical techniques, limited sample sizes, and potential confounding from co-exposure to other environmental pollutants such as heavy metals, persistent organic pollutants, and other endocrine-disrupting chemicals. Despite these limitations, accumulating evidence from biomonitoring and exposure assessment studies highlights the pervasive presence of MP/NPs in human tissues, suggesting that chronic low-dose exposure is widespread. Future epidemiological studies, particularly large prospective cohort studies with adequate sample sizes, standardized exposure measurements, and rigorous adjustment for confounding variables, are essential to strengthen the evidence base. These approaches will improve causal inference and enhance the external validity of findings regarding the potential role of MP/NP exposure in reproductive cancer development and progression.

## 3. Anticancer Properties of Ginger, Turmeric, and Garlic

Phytochemicals have gained increasing attention in oncology due to their biocompatibility and diverse pharmacological properties. These naturally occurring compounds, abundant in plants, are being explored for their potential to prevent and manage various cancers. The Sub-Saharan and Mediterranean regions, in particular, are endowed with a rich diversity of medicinal plants traditionally used for health promotion, many of which exhibit promising anticancer effects [81,82]. Among the plant-derived sources, culinary spices are unique because of their dual role in enhancing food flavor and contributing to disease prevention [83]. Numerous studies have reported the antioxidant, anti-inflammatory, and immunomodulatory activities of spices, suggesting that their regular dietary inclusion may lower cancer risk [84,85]. Traditionally, spices form an essential part of regional cuisines, used in soups, roasted meats, fish dishes, marinades, rice, salads, dumplings, jams, and marmalades. Their widespread use in everyday meals provides an accessible means of delivering bioactive compounds with potential therapeutic value.

Among these spices, ginger (*Zingiber officinale*) has received significant scientific attention. It is widely appreciated for its distinctive aroma, pungent taste, and carminative qualities, making it a common ingredient in numerous traditional recipes [86]. Based on chemical composition, ginger is rich in phenolic compounds, terpenes, polysaccharides, lipids, organic acids, and fibers [87]. Importantly, its phenolic constituents, especially gingerols and shogaols, have been identified as key contributors to its antioxidant, anti-inflammatory, and anticancer activities [88,89]. Also, turmeric (*Curcuma longa* L.), a spice native to India and a fundamental component of curry powder, has been extensively investigated for its bioactive potential [90]. The health-promoting properties of turmeric are primarily attributed to its curcuminoids, like curcumin, demethoxycurcumin, and bisdemethoxycurcumin [91]. These compounds possess potent antioxidant and anti-inflammatory effects that underpin turmeric’s growing recognition as a promising natural agent in cancer prevention and adjunct therapy [92].

Similarly, garlic (*Allium sativum*) is another globally valued spice with well-documented medicinal applications. Consumed in various forms (powder, or paste), garlic is esteemed not only for its characteristic flavor but also for its therapeutic benefits [93]. Historical evidence from ancient Egypt and Greece indicate its use to enhance strength and protect against diseases, reflecting its long-standing medicinal relevance [94]. Garlic owes its biological activity mainly to sulfur-containing compounds, particularly sulfenic acids that form thiosulfonates [95]. Among these, allicin is the most abundant and pharmacologically active compound, widely recognized for its antimicrobial, antioxidant, and anticancer properties.

### 3.1. Bioactive Compounds in Ginger, Turmeric, and Garlic

**Figure 4:**
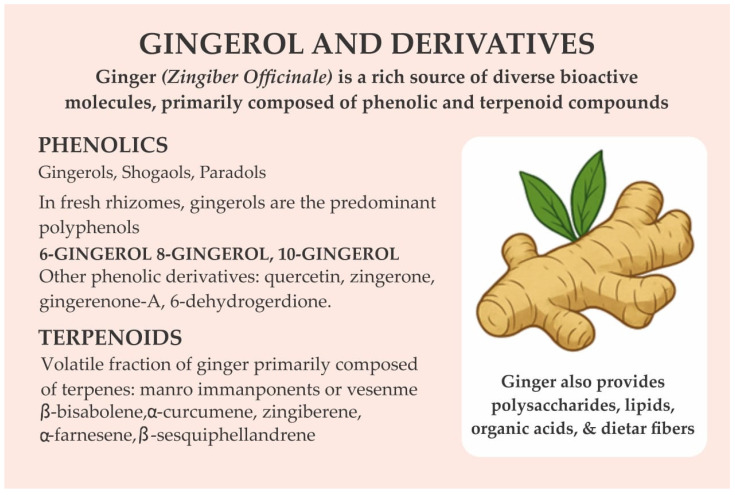
Gingerol and Its Major Bioactive Derivatives; This figure illustrates the key classes of bioactive compounds present in ginger, including phenolic constituents such as gingerols, shogaols, and paradols, and their predominant forms (e.g., 6-gingerol, 8-gingerol, 10-gingerol). Additional phenolic derivatives like quercetin, zingiberone, gingerenone-A, and 6-dehydrogingerdione are also highlighted. Ginger’s terpenoid fraction is composed of compounds such as β-bisabolene, α-curcumene, zingiberene, α-farnesene, and β-sesquiphellandrene. Also, ginger provides polysaccharides, lipids, organic acids, and dietary fibers.

Ginger is a rich source of diverse bioactive molecules, primarily composed of phenolic and terpenoid compounds. The major phenolics include gingerols, shogaols, and paradols, which collectively contribute to its characteristic pungency and biological activity [96]. In fresh rhizomes, gingerols, notably 6-gingerol, 8-gingerol, and 10-gingerol, are the predominant polyphenols [97]. During heat processing or prolonged storage, these compounds undergo dehydration reactions, yielding shogaols, while subsequent hydrogenation can convert shogaols into paradols [98]. In addition to these key constituents, ginger also contains other phenolic derivatives such as quercetin, zingerone, gingerenone-A, and 6-dehydrogingerdione [99]. Ginger’s volatile fraction is primarily composed of terpenes, including β-bisabolene, α-curcumene, zingiberene, α-farnesene, and β-sesquiphellandrene, which form the major components of its essential oil [99]. Beyond these, ginger provides polysaccharides, lipids, organic acids, and dietary fibers, all of which contribute to its nutritional and pharmacological properties (Figure 4).

**Figure 5:**
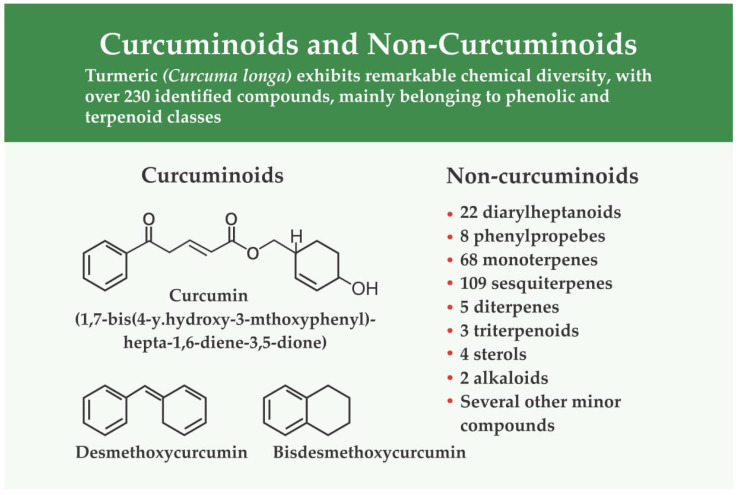
Curcumoids and Non-Curcumoids Constituents of Turmeric; this figure presents the major phytochemical groups derived from *Curcuma longa*. The curcuminoid class, represented by curcumin, demethoxycurcumin, and bisdemethoxycurcumin, is highlighted along with their characteristic chemical structures. Turmeric contains a much broader array of non-curcuminoid compounds, including diarylheptanoids, phenylpropene derivatives, monoterpenes, sesquiterpenes, diterpenes, triterpenoids, sterols, alkaloids, and several minor constituents.

Turmeric exhibits remarkable chemical diversity, with over 230 identified compounds, mainly belonging to phenolic and terpenoid classes [90,100]. Among these, curcuminoids represent the most studied bioactive constituents. These are diarylheptanoids, characterized by two aromatic rings linked by a seven-carbon chain (Figure 5). The principal curcuminoid, curcumin (diferuloylmethane), is accompanied by desmethoxycurcumin and bisdesmethoxycurcumin [101]. Structurally, curcumin (1,7-bis(4-hydroxy-3-methoxyphenyl)-hepta-1,6-diene-3,5-dione) is a lipophilic polyphenol, and its hydrophobic aromatic rings enable effective interaction with biological membranes and molecular targets [102]. Apart from curcuminoids, turmeric also contains 22 diarylheptanoids, 8 phenylpropenes, 68 monoterpenes, 109 sesquiterpenes, 5 diterpenes, 3 triterpenoids, 4 sterols, 2 alkaloids, and several other minor compounds, all contributing to its broad pharmacological potential [103].

**Figure 6:**
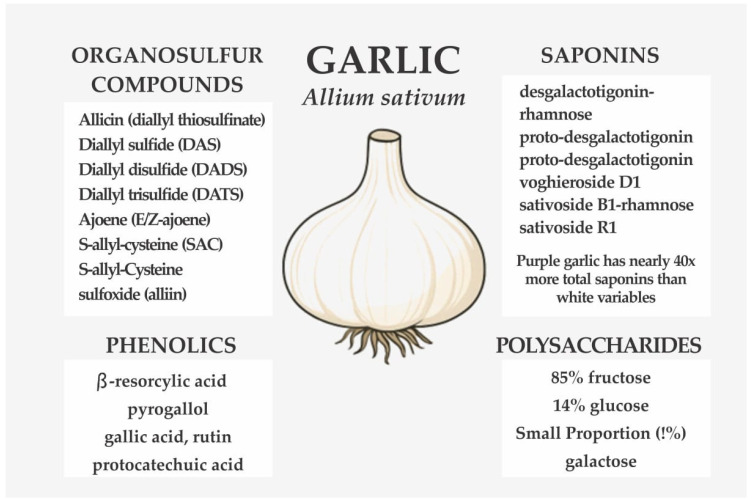
Organosulfur compounds, phenolics, saponins, and other components of garlic.

Garlic contains a wide range of bioactive constituents, notably organosulfur compounds, saponins, phenolics, and polysaccharides [104]. The organosulfur compounds, including allicin (diallyl thiosulfinate), diallyl sulfide (DAS), diallyl disulfide (DADS), diallyl trisulfide (DATS), ajoene (E/Z-ajoene), S-allyl-cysteine (SAC), and S-allyl-cysteine sulfoxide (alliin)*,* represent the principal active metabolites responsible for most of garlic’s biological effects [105]. These compounds are generally more bioavailable in raw garlic than in cooked forms, where thermal degradation can occur [105]. In contrast, saponins are relatively heat-stable and vary with garlic type; purple garlic, for instance, possesses nearly forty times more total saponins than white varieties and uniquely contains compounds such as desgalactotigonin-rhamnose, proto-desgalactotigonin, proto-desgalactotigonin-rhamnose, voghieroside D1, sativoside B1-rhamnose, and sativoside R1 [106]. Garlic also contains an abundance of phenolic compounds, with more than 20 identified (Figure 6). The β-resorcylic acid is the most abundant, followed by pyrogallol, gallic acid, rutin, protocatechuic acid, and quercetin. Additionally, garlic polysaccharides consist of fructose (85%)*,* glucose (14%)*,* and a small proportion of galactose (1%) [107].

### 3.2. Molecular Mechanisms of Bioactive Compounds

#### 3.2.1. Evidence from Preclinical Studies

##### Ginger

Several studies have explored the cytotoxic and antiproliferative effects of ginger-derived compounds, particularly 6-gingerol and 10-gingerol, on various cancer cell types through distinct molecular mechanisms. In colon cancer cells, 10-gingerol was shown to exert cytotoxicity by activating the mitogen-activated protein kinase (MAPK) signaling pathway in a dose-dependent manner [89]. Similarly, 6-gingerol demonstrated antiproliferative activity in human keratinocyte and mouse skin tumor cell lines by modulating MAPK and AP-1 signaling pathways, as well as activating NF-κB, p38 MAPK, and cyclooxygenase-2 (COX-2) expression [108].

In an oral cancer-related study, 6-shogaol suppressed cell proliferation and migration by inhibiting the AKT/mTOR signaling pathway and activating AMP-activated protein kinase (AMPK), ultimately leading to cell cycle arrest and apoptosis [98]. Likewise, in gastric cancer, 6-gingerol enhanced the cytotoxic efficacy of cisplatin chemotherapy by modulating the PI3K/AKT pathway, resulting in G1-phase cell cycle arrest [109]. Additional evidence indicates that 6-gingerol can also induce G2-phase arrest in cervical carcinoma cells, suggesting a cell-type-specific regulatory effect on cell cycle checkpoints [110]. Studies on renal carcinoma cells revealed that 6-gingerol triggered G1-phase arrest [111], reinforcing its role in suppressing tumor growth in multiple organ systems. Similar findings were observed in osteosarcoma, where 6-gingerol inhibited cell proliferation through AMPK pathway activation, leading to G1-phase arrest and growth inhibition [112].

Further studies identified that 6-gingerol can inhibit pancreatic cancer cell growth by downregulating the ERK/NF-κB/Snail signaling axis, highlighting another route by which ginger components exert anti-metastatic and pro-apoptotic effects [113]. In renal cell carcinoma models, both in vitro and in vivo experiments have shown that 6-gingerol suppresses tumor metastasis by enhancing YAP (Yes-associated protein) ser127 phosphorylation and reducing nuclear YAP accumulation [114], mechanisms that collectively limit cancer cell migration and invasion. Endometrial cancer remains one of the most prevalent malignancies affecting the female reproductive system. Among natural bioactive compounds, 6-shogaol, a pungent phenolic constituent derived from ginger, has been shown to possess anticancer activity against human endometrial carcinoma cells [115]. This compound suppresses cell proliferation by inducing cell cycle arrest at the G2/M phase, and promotes apoptotic cell death along with elevated level of reactive oxygen species.

##### Turmeric

Curcumin, the principal bioactive compound of turmeric, has been extensively studied for its broad anticancer potential. It exhibits inhibitory activity at multiple stages of carcinogenesis, including tumor initiation, promotion, progression, angiogenesis, and metastasis. Mechanistically, curcumin regulates diverse molecular targets that govern cell proliferation, apoptosis, and invasion. It downregulates cyclin D1 and c-myc in the proliferation pathway, suppresses anti-apoptotic proteins such as Bcl-2, Bcl-xL, cFLIP, and activates the caspase cascade involving caspase-8, -3, and -9 [116]. Additionally, it promotes tumor suppressor proteins (p53 and p21), activates death receptors (DR4 and DR5), and modulates signaling cascades such as JNK, Akt/PKB, and AMPK [117]. These molecular interactions explain the wide-ranging anticancer effects attributed to curcumin and its derivatives.

Building upon these molecular insights, several studies have compared the biological efficacy of curcuminoid analogs like curcumin (CUR), demethoxycurcumin (DMC), bisdemethoxycurcumin (BDMC), and cyclocurcumin across different cancer models [32]. In MCF-7 human breast cancer cells, DMC showed greater antiproliferative activity than CUR and BDMC, a property linked to the presence of phenolic hydroxyl and methoxyl groups, as well as the diketone moiety [118,119]. These findings emphasize that subtle structural variations among curcuminoids can significantly influence their bioactivity and therapeutic potential.

In addition, curcumin has been shown to exert broader biological activities that may complement its anticancer action. For instance, Jiang et al. identified curcuminoids from *turmeric* as active antitumor agents against HeLa cells [120]. Such findings highlight the versatility of curcumin’s biological effects, bridging metabolic regulation with cancer prevention.

The influence of curcuminoids on tumor invasion and metastasis has also been well characterized. Yodkeeree et al. compared the effects of CUR, DMC, and BDMC on the expression of urokinase plasminogen activator (uPA), matrix metalloproteinases (MMPs), membrane type 1-MMP (MT1-MMP), and tissue inhibitors of metalloproteinases (TIMPs) in human fibrosarcoma cells [121]. Their findings revealed a potency order of BDMC > DMC > CUR in reducing invasive potential.

##### Garlic

**Figure 7:**
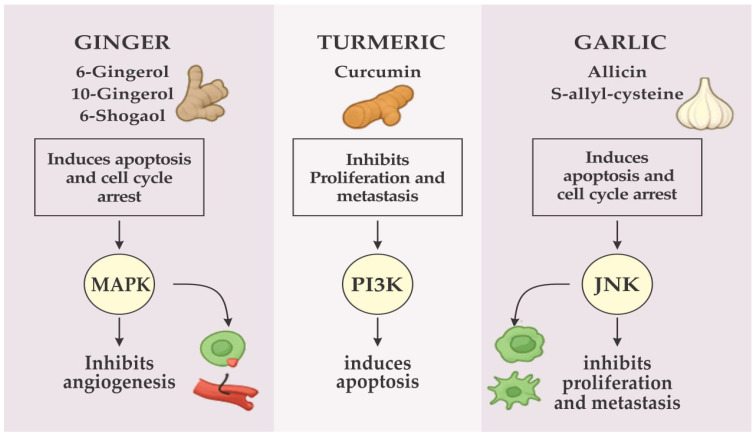
Summary of the mechanism of action of ginger, turmeric, and garlic.

The anticancer activity of garlic and its bioactive derivatives is mediated by multiple mechanisms, including ROS generation, regulation of PI3K/Akt, JNK, and STAT3 pathways, inhibition of angiogenesis, and metastasis [122,123]. Black garlic and its bioactive derivatives exhibit multifaceted anticancer properties mediated through diverse molecular pathways that regulate oxidative stress, apoptosis, and cell proliferation. Central to these mechanisms is the modulation of redox-sensitive signaling cascades, particularly the activation of c-Jun N-terminal kinase (JNK) by reactive oxygen species (ROS) [124] (Figure 7). The resultant oxidative stress downregulates anti-apoptotic proteins such as MCL-1 and BCL-2 while enhancing the expression of pro-apoptotic factors BIM and BAK [125]. These molecular events culminate in apoptotic cell death and reduced proliferation in estrogen receptor-positive (ER+) breast cancer cell lines, including MCF-7 and MDA-MB-361 [126].

Allicin, another prominent sulfur compound in garlic, exerts concentration-dependent anticancer effects through various mechanisms. In gastric cancer cells (AGS, HGC27), allicin induces intrinsic apoptosis by upregulating miR-383-5p and ERBB4 while suppressing the PI3K/Akt pathway [127]. Beyond apoptosis, allicin also induces cell cycle arrest and inhibits metastatic behavior. In multiple cancer models, including leukemia (HL-60), colorectal (Caco-2), and lung (A549) cells, allicin promotes G2/M phase arrest through oxidative-stress-mediated DNA damage and p53 activation [128]. Additionally, it suppresses migration, invasion, and angiogenesis by blocking VEGF-induced endothelial proliferation and tube formation [129].

### 3.3. Evidence from Clinical Trials

Recent clinical findings have shown that bioactive compounds derived from turmeric, ginger, and garlic possess potential therapeutic and adjuvant effects against reproductive and other forms of cancers, although conclusive proof of efficacy in humans remains limited (Table 2). Among all these agents, curcumin has been reported to show the most consistent results in early-phase clinical trials targeting breast [130], pancreatic [131], and colorectal [132]. Many pilot and Phase I/II studies have shown that curcumin can modulate inflammatory and apoptotic biomarkers, enhance chemoradiosensitivity, and improve treatment tolerance (Table 2). For instance, two ongoing clinical trials (e.g., NCT04294836, NCT05947513) have evaluated the potential adjuvant therapeutic role of nanoformulated curcumin (NEC) in patients with prostate cancer [133] and bladder cancer [134]. They reported the favorable effects of nanoformulated curcumin therapy on oxidative and inflammatory parameters. The clinical role of ginger in reproductive oncology appears to be supportive. The direct anticancer activity in humans has not been confirmed, but randomized controlled trials and meta-analyses in breast cancer populations have shown significant reductions in chemotherapy-induced nausea and vomiting [135,136]. In contrast, the reports on the anticancer effect of garlic in reproductive malignancies were largely deduced from epidemiological associations [137,138,139], suggesting reduced prostate, breast, and cervical cancer risks among individuals with high dietary intake. However, interventional trials remain scarce and inconclusive, and different garlic preparation methods complicate clinical interpretation. Finally, despite the promising adjunctive and preventive potential of these phytochemicals, the current clinical evidence is limited by small sample sizes, heterogeneity of formulations, and reliance on surrogate endpoints. Future research should prioritize standardized formulations, mechanistically informed biomarkers, and adequately powered randomized trials to substantiate their therapeutic utility in reproductive cancers.

### 3.4. Synergistic Effects and Enhanced Bioavailability Strategies

Emerging evidence from preclinical studies suggests that the combined use of ginger, turmeric, and garlic, and their respective bioactive compounds (gingerols and shogaols, curcuminoids, and organosulfur compounds), exerts a more potent effect against reproductive cancers than when used individually [91,140,141]. Sulfur-containing compounds and polyphenols present in ginger, turmeric, and garlic have been shown to interfere with diverse signaling cascades involved in the initiation and progression of reproductive organ cancers. The co-administration of ginger and turmeric extracts has been reported to modulate key oncogenic pathways, including NF-κB, PI3K/Akt/mTOR, and MAPK, thereby inducing apoptosis in breast and cervical cancer cells [142,143]. Allicin derivatives from garlic further enhance anticancer efficacy by promoting oxidative stress and mitochondrial-mediated cell death, complementing the anti-angiogenic and anti-metastatic actions of curcumin and 6-gingerol [144]. Preclinical studies also indicate that combined extracts of turmeric, ginger, and garlic exert synergistic inhibition of estrogen receptor (ER) signaling in breast cancer and androgen receptor (AR) signaling in prostate cancer [145,146]. Collectively, the ability of these phytochemicals to cooperatively modulate multiple reproductive cancer pathways highlights their potential as dietary adjuvants or complementary therapeutic agents.

The clinical efficacy of curcumin, gingerol, and allicin is limited by their poor solubility, chemical instability, and rapid metabolism (Table 3). To overcome these challenges, several bioavailability-enhancing strategies have been developed: (i) co-administration of curcumin and gingerol with piperine to inhibit glucuronidation and efflux transporters, thereby increasing their systemic retention [147,148]; (ii) conjugation or co-formulation with liposomal and phospholipid-based nanoparticles to improve solubility, controlled release, and cellular uptake [149]; and (iii) encapsulation of garlic extracts to stabilize and preserve allicin bioactivity during gastrointestinal digestion [150]. The co-formulation of these bioactives into nanoemulsions or functional food matrices has been shown to enhance systemic exposure while maintaining their synergistic anticancer efficacy against reproductive tumors.

## 4. Conceptual Model: Potential Modulatory Role of Dietary Spices in MP/NP-Induced Carcinogenic Pathways

Although the biological pathways discussed in this review suggest a plausible link between MP/NP exposure and reproductive carcinogenesis, it is important to emphasize that direct experimental or epidemiological studies examining the interaction between MP/NP exposure, dietary spices, and cancer risk are currently lacking. Consequently, the potential protective roles of bioactive compounds derived from dietary spices should be interpreted within a hypothesis-driven conceptual framework based on shared molecular pathways, rather than as evidence of a confirmed causal relationship.

Prolonged exposure to MP/NPs has been reported to induce excessive production of ROS and RNS, resulting in oxidative stress. Persistent oxidative stress is widely recognized as a key contributor to inflammation, genomic instability, and carcinogenic processes [157]. Oxidative imbalance is also closely associated with cellular senescence, which contributes to cumulative cellular damage, cell-cycle arrest, and increased susceptibility to carcinogenic transformation [158]. At the cellular level, accumulation of MP/NPs may promote ROS overproduction, leading to lipid peroxidation, protein oxidation, mitochondrial dysfunction, and oxidative DNA damage, all of which can disrupt normal cellular homeostasis.

**Figure 8:**
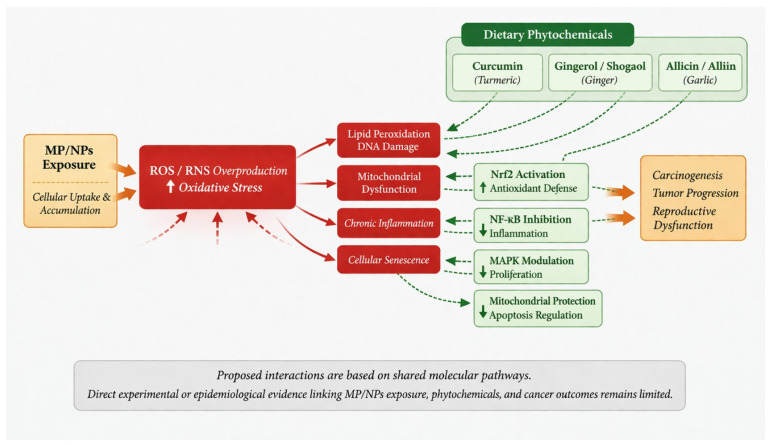
Hypothesized Interactions Between MP/NPs-Induced Cellular Stress and Phytochemical Anticancer Pathways. This conceptual framework depicts proposed biological pathways linking chronic exposure to micro- and nanoplastics (MP/NPs) with cellular stress responses and potential carcinogenic effects in reproductive systems, highlighting possible modulatory roles of dietary phytochemicals. MP/NPs may be internalized by cells, triggering excessive reactive oxygen and nitrogen species (ROS/RNS) production, oxidative stress, and downstream molecular damage, including lipid peroxidation, protein oxidation, DNA lesions, mitochondrial dysfunction, and apoptosis dysregulation. These events activate inflammatory and stress–response pathways (e.g., NF-κB, MAPK), induce senescence, and promote a pro-tumorigenic microenvironment. The figure also illustrates potential intervention points of bioactive compounds from turmeric (curcumin), ginger (gingerol, shogaol), and garlic (allicin, alliin), which may exert antioxidant, anti-inflammatory, and cytoprotective effects through Nrf2 activation, NF-κB inhibition, MAPK modulation, and regulation of mitochondrial and apoptotic pathways.

Given that many commonly consumed spices contain bioactive phytochemicals with antioxidant and anti-inflammatory properties, it is plausible that these compounds could modulate some of the molecular pathways activated during MP/NP-induced cellular stress. For example, turmeric, ginger, and garlic contain compounds such as curcumin, gingerol/shogaol, and allicin/alliin, respectively. These phytochemicals have been widely reported to regulate cellular defense mechanisms by activating cytoprotective pathways such as nuclear factor erythroid 2–related factor 2 (Nrf2), inhibiting nuclear factor-κB (NF-κB)-mediated inflammatory signaling, and modulating mitogen-activated protein kinase (MAPK) pathways. Through these mechanisms, such compounds may theoretically reduce oxidative damage, modulate inflammatory responses, and help maintain cellular homeostasis under conditions of environmental stress (Figure 8).

Emerging experimental studies have begun to explore the potential protective effects of certain phytochemicals against plastic-related toxicity. For instance, curcumin has been shown in experimental models to exert tissue-protective effects against environmental toxicants through modulation of oxidative and inflammatory signaling pathways. Preclinical studies suggest that curcumin may mitigate MP/NP-induced toxicity in several organ systems by restoring redox balance and suppressing stress-related molecular cascades. Mechanistically, curcumin has been reported to reduce ROS generation, enhance antioxidant defenses through activation of the Nrf2/HO-1 pathway, and attenuate inflammatory signaling via inhibition of NF-κB and MAPK pathways [159]. In addition, curcumin has been shown to regulate mitochondrial integrity and apoptosis-related signaling pathways, including modulation of Bax, Bcl-2, and caspase-3, thereby reducing oxidative damage and inflammatory responses in experimental models [160,161]. However, it is important to note that most of these findings derive from controlled experimental systems, and their direct relevance to human MP/NP exposure is yet to be established.

Evidence supporting the protective effects of ginger and garlic is also largely derived from experimental studies involving other environmental toxicants, including bisphenol A (BPA), phthalates, arsenic, and heavy metals [162]. In these models, both ginger- and garlic-derived compounds demonstrate antioxidant, anti-inflammatory, and cytoprotective activities. Because many of the molecular pathways activated by these toxicants, including ROS generation, NF-κB signaling, mitochondrial dysfunction, and cytokine-mediated inflammation, overlap those implicated in MP/NP-induced toxicity, these observations provide a mechanistic rationale for hypothesizing that similar protective interactions may occur in the context of plastic-particle exposure.

### Research Challenges

Studies investigating the reproductive toxicity of MP/NPs have advanced substantially, yet they remain constrained by significant methodological and interpretative challenges. A central issue is the pronounced heterogeneity of MP/NPs in terms of particle size, shape, polymer composition, and surface chemistry, which complicates cross-study comparisons and findings. Most preclinical research relies on polystyrene microspheres, a simplification that poorly reflects real-world environmental exposures, where MP/NPs are chemically diverse and frequently carry additives or adsorbed environmental pollutants. This lack of environmental relevance intersects with exposure assessment challenges, collectively limiting translational validity.

A related concern involves the evaluation of realistic exposure doses and durations. Many experimental models employ MP/NP concentrations that substantially exceed those encountered in everyday human exposure, thereby constraining the extrapolation of preclinical outcomes to real-world risk scenarios [163]. Moreover, humans are primarily exposed to MP/NPs via oral ingestion, inhalation, and dermal contact, each of which exhibits distinct absorption efficiencies, biodistribution patterns, and clearance dynamic [26]. Despite this, comparative analyses of exposure-route-specific kinetics are scarce, and data on bioaccumulation processes, such as tissue retention, degradation, and excretion, remain limited. These gaps hinder accurate modeling of internal exposure and long-term reproductive risk.

Compounding these issues, the quantification of MP/NPs in human tissues poses substantial technical challenges. High risks of background contamination, coupled with the absence of standardized analytical protocols, undermine measurement reliability [164]. Variability in sample preparation procedures, detection limits, and particle identification methods contributes to inconsistent exposure estimates across studies. Standardization of analytical techniques is therefore essential to improve reproducibility, facilitate interstudy comparisons, and support meaningful dose–response assessments.

Another critical and intersecting challenge lies in disentangling the intrinsic toxicological effects of MP/NPs from those mediated by associated chemicals. MP/NPs can function as vectors for hydrophobic organic pollutants and heavy metals, potentially enhancing, attenuating, or qualitatively altering toxicological responses. In biological environments, MP/NPs also interact with proteins, lipids, and other biomolecules to form a dynamic “corona,” which can influence particle stability, cellular uptake, and biological reactivity [165]. Despite its likely relevance to reproductive toxicity, this phenomenon remains insufficiently characterized within reproductive systems and developmental contexts.

Addressing these interrelated limitations requires integrative research frameworks that bridge in vitro, in vivo, and epidemiological evidence while incorporating environmentally realistic exposure scenarios, interspecies variability, and long-term outcome assessment. The development of physiologically based pharmacokinetic (PBPK) models, alongside the application of omics-based approaches, holds promise for elucidating underlying molecular mechanisms and enhancing translational relevance. Ultimately, resolving these methodological and conceptual challenges will be critical for robust risk assessment and for establishing causal links between MP/NP exposure and reproductive pathologies.

## 5. Public Health Perspectives and Future Research Directions

Dietary fortification with bioactive spices such as turmeric, ginger, and garlic can serve as chemoprotective agents against reproductive cancer. The curcuminoids in turmeric, gingerols and shogaols in ginger, and organosulfur compounds in garlic have all shown free radicals scavenging capacities, suppress NF-κB-mediated inflammation, activate Nrf2/HO-1 antioxidant signaling, and restore mitochondrial function, all of which are key mechanisms of MP/NP-induced toxicity. Consistent addition of these spices in culinary practices, at physiologically relevant dietary doses, may therefore offer sustained protective modulation against MP/NP-induced oxidative and inflammatory insults to body tissue. Notably, synergistic consumption (e.g., ginger–turmeric blends) may provide enhanced bioactivity through complementary mechanisms targeting multiple redox and apoptotic pathways.

As discussed in the previous section, early-phase clinical data on nanoformulated curcumin and aged garlic extract revealed positive effects against oxidative and inflammatory biomarkers without significant adverse effects, supporting their suitability as nutraceutical interventions in high-risk populations. However, supplementation should be contextualized within overall dietary quality and lifestyle. Over-reliance on isolated extracts without concurrent modification of exposure sources and dietary habits may yield limited benefit. Thus, integrative nutritional approaches that combine MP/NP exposure reduction, antioxidant-rich diets, and controlled supplementation appear most promising.

From a translational perspective, promoting dietary diversification and the inclusion of culturally acceptable spice-based functional foods represents a cost-effective, population-level strategy to mitigate emerging plastic-associated health risks, particularly those linked to reproductive cancers. Public health nutrition initiatives should prioritize education on safe food storage practices, advocate the regular consumption of antioxidant- and anti-inflammatory-rich spices, and support well-designed clinical trials to evaluate the long-term efficacy of such interventions. Although targeted interventions for MP/NP-induced carcinogenic risk are still in early development, current evidence underscores dietary modification, especially fortification with bioactive spices, as a feasible preventive approach consistent with global health and sustainability objectives.

Despite growing recognition of the potential health risks associated with micro- and nanoplastics (MP/NPs) and the promising protective roles of bioactive dietary components, critical research gaps persist. Future research should make the development and validation of standardized methodologies for detecting and quantifying MP/NPs in biological and environmental samples a priority. Recent inconsistencies in particle size characterization, polymer identification, and contamination control are hindering data comparison and limiting exposure–response assessments. Harmonized analytical protocols employing advanced spectroscopic and imaging technologies will improve accuracy in exposure quantification and enable cross-study integration.

Although oxidative stress, inflammation, and endocrine disruption are established pathways, the exact molecular interactions between *MP/NPs* and reproductive tissues are not completely understood. Future studies should employ transcriptomic, proteomic, metabolomic, and epigenomic profiling to uncover the signaling networks and gene–environment interactions underlying MP/NP-induced carcinogenesis. Systems biology and computational modeling can further delineate how chronic low-dose exposure influences cellular homeostasis and tumorigenic transformation. Most of the preclinical studies depend on high-dose and short-term exposures that poorly mimic human environmental conditions. Future in vivo and in vitro models should adopt environmentally relevant concentrations, exposure durations, and mixed-polymer systems that better reflect human exposure scenarios. Animal studies and prospective human cohorts integrating environmental, dietary, and molecular biomarkers will be crucial to the establishment of the relationships between MP/NP exposure and reproductive cancer risk.

Although preclinical evidence supports the chemoprotective effects of ginger, turmeric, and garlic, clinical data remain limited. Well-powered randomized controlled trials using standardized, bioavailable formulations of these spices are needed to confirm efficacy and determine optimal dosing regimens. Translational research should also focus on developing spice-based functional foods and nutraceutical formulations that are culturally acceptable, cost-effective, and easily integrated into daily diets, especially in high-risk or resource-limited settings. Human exposure rarely occurs in isolation. Individuals are simultaneously exposed to complex mixtures of MPs, NPs, and associated plastic additives, often alongside other environmental contaminants. Future investigations should therefore explore combined and synergistic toxicological effects, as well as how dietary antioxidants and micronutrient status may modulate these interactions. Such data will be critical for refining risk assessment models and guiding nutritional intervention strategies.

Integrating scientific evidence with public health and environmental policy will be crucial for effective prevention. Research should inform policy frameworks that promote plastic reduction, safe food packaging, and dietary interventions emphasizing natural antioxidant sources. Collaborative programs linking environmental surveillance, nutrition education, and sustainable agriculture will strengthen the translational impact of MP/NP-related findings and align mitigation strategies with the United Nations Sustainable Development Goals (SDGs).

## Figures and Tables

**Table 1:** Human Biomonitoring Evidence of Microplastics in Biological Samples.

Biological Matrix	Key Findings	Potential Health Implications	References
Blood	MP/NP particles detected in circulating human blood samples, suggesting systemic distribution following environmental exposure.	Indicates the ability of MP/NPs to circulate throughout the body and potentially reach multiple organs, including reproductive tissues.	[61]
Placental Tissue	MP/NPs identified in placental samples from healthy pregnancies.	Suggests possible maternal–fetal transfer and potential effects on fetal development and reproductive health.	[63]
Lung Tissue	Presence of microplastics detected in human lung tissues obtained during surgical procedures.	Supports inhalation as a major route of exposure and indicates possible respiratory and systemic health risks.	[64]
Ovarian Follicular Fluid	MP/NPs detected in follicular fluid collected from women undergoing assisted reproductive procedures.	It indicates potential direct interaction with oocytes and reproductive cells, raising concerns regarding fertility and reproductive toxicity.	[19]
Human Feces	MP/NP s consistently detected in stool samples across different populations.	Confirms widespread dietary exposure and gastrointestinal uptake of plastic particles.	[65]

**Table 2:** Summary of clinical evidence of turmeric (curcumin), ginger, and garlic in reproductive cancers.

Compound	Cancer Type	Study Category	Study Design/Phase	Sample Size	Outcome/Parameters	Key Findings	Reference/Trial ID	Status
Curcumin (turmeric)	Breast cancer	Clinical trial	Pilot/Phase II	Small cohort (*n* < 100)	Oxidative stress markers, inflammatory biomarkers, treatment tolerability	Improved oxidative and inflammatory biomarkers; enhanced tolerance to chemo/radiotherapy	Alliance A22_Pilot9	Completed
Curcumin	Cervical cancer	Clinical trial	Phase I/II	Not reported (early-phase)	Apoptotic gene expression, radiosensitivity	Enhanced apoptosis and radiosensitization	NCT04294836, NCT05947513	Ongoing
Curcumin	Prostate cancer	Clinical trial	Early-phase (pilot biomarker study)	Small cohort	PSA levels, oxidative stress biomarkers	Modulation of PSA and oxidative stress markers	[133]	Completed
Curcumin	Ovarian cancer	Exploratory/adjunct	Translational/adjunct study	Limited (small-scale)	Chemosensitivity (platinum response), biomarkers	Preliminary evidence of enhanced chemosensitivity	Small-scale adjunct studies	Completed/Ongoing
Ginger (*Zingiber officinale*)	Breast cancer	Clinical trial	Randomized controlled trials (RCTs)	Moderate (varies across RCTs)	Chemotherapy-induced nausea and vomiting (CINV), quality of life	Significant reduction in CINV; improved QoL; no direct tumor response evidence	[135,136]	Completed
Ginger	Ovarian/Cervical/Prostate	Exploratory/preclinical	Preclinical/biomarker-based	Not applicable	Molecular and symptom-related endpoints	No human clinical evidence for anticancer efficacy	—	—
Garlic (*Allium sativum*)	Prostate cancer	Exploratory/small-scale	Small interventional/observational	Small cohorts	PSA levels, epidemiological associations	Mixed findings; inconsistent PSA modulation	Early clinical/epidemiological studies	Mixed
Garlic	Breast, Cervical, Ovarian	Observational	Population-based/dietary studies	Large population datasets	Cancer incidence/risk	Higher intake associated with reduced risk; causality not established	Meta-analysis, cohort studies	Completed

**Table 3:** Bioactive Compounds, Mechanisms, and Bioavailability Enhancement Strategies in Reproductive Cancers.

Plant (Common Name)	Major Bioactive Compounds	Targeted Reproductive Cancers	Mechanistic Actions	Bioavailability Challenges	Enhancement Strategies
*Curcuma longa* (Turmeric)	Curcumin, Demethoxycurcumin	Breast, Cervical, Ovarian, Prostate	Inhibits NF-κB, PI3K/Akt, induces apoptosis, suppresses angiogenesis	Poor solubility, rapid metabolism	Piperine co-administration, nanoparticles, liposomes [151,152]
*Zingiber officinale* (Ginger)	6-Gingerol, 6-Shogaol	Breast, Ovarian, Prostate	Induces apoptosis, inhibits cell migration/invasion, modulates p53 and Bax/Bcl-2	Low stability, poor absorption	Lipid nanoparticles, nanoemulsions, and proliposomes [153,154]
*Allium sativum* (Garlic)	Allicin, S-allyl cysteine	Cervical, Prostate, Breast	Triggers ROS-mediated apoptosis, suppresses AR/ER signaling, and enhances immune modulation	Instability of allicin, degradation in the GI tract	Encapsulation, aged garlic extract stabilization [150,155]
Combined Formulation	Mixed bioactives	Breast, Cervical, Prostate	Synergistic inhibition of ER/AR signaling, NF-κB suppression, and enhanced apoptosis	Variable pharmacokinetics	Nanoformulated blends, piperine-based adjuvants [156]

## Data Availability

Data sharing is not applicable to this article as no new data were created or analyzed in this study.
